# The taxonomy of *Amycolatopsis lurida* TRM64739 and *Bacillus haynesii*

**DOI:** 10.3389/fmicb.2025.1571458

**Published:** 2025-05-23

**Authors:** Zhan-wen Liu, Xin-rong Luo, Zhan-feng Xia, Chuan-xing Wan, Li-li Zhang

**Affiliations:** ^1^State Key Laboratory Incubation Base for Conservation and Utilization of Bio-Resource in Tarim Basin, Tarim University, Alar, China; ^2^College of Life Sciences and Technology, Tarim University, Alar, China

**Keywords:** genome mining, co-culture, *Amcolatopsis lurida* TRM64739, *Bacillus haynesii*, phenazine compounds

## Abstract

**Introduction:**

Actinomycetes are a significant source of natural products. *Amycolatopsis*, a rare actinomycete, is particularly noted for its robust potential in secondary metabolite production, but most of the biosynthetic gene clusters (BGCs) are silent. To discover new BGCs and their metabolites, this study employed genome mining and co-culture techniques to explore the secondary metabolites produced by *Amycolatopsis lurida* TRM64739.

**Method:**

A novel BGC was identified in A.lurida TRM64739 using antiSMASH and phylogenetic analysis, this new BGC was activated through co-culturing *A. lurida* TRM64739 with *Bacillus haynesii*. Subsequently, a series of phenazine compounds were isolated and identified by chromatographic separation, such as silica gel column (100-200 mesh), Sephadex LH-20 and HPLC, and spectral analysis, such as NMR and UPLC-HRESI-MS/MS, respectively.

**Results:**

Five phenazine compounds were isolated and identified as compound 1(1,6-Dimethoxyphenazine), compound 2(1,6-Dihydroxyphenazine), compound 3(phenazine-1-carboxylic acid), compound 4(6-hydroxy-1-methoxyphenazine), and compound 5(1,6-p-chlorophenylphenazine). Among these, compounds1-4 are known, while compound 5(1,6-p-chlorophenylphenazine) represents a new compound and has exhibited antimicrobial activity to clinically drug-resistant strains (*A. baumannii* ATCC19606, *P. aeruginosa* ATCC27853) and plant pathogenic bacteria (*E. amylovora* ATCC BAA-2158).

**Discussion:**

Our work also demonstrates that the combined approach of genome mining and activation of silent BGCs is a useful method for the discovery of new natural products.

## 1 Introduction

Natural products, particularly those derived from microbial sources, are extensively utilized in agriculture, medicine, veterinary medicine, and other fields, providing significant benefits to society (Abdel-Razek et al., 2020; Atanasov et al., 2021). However, the widespread use of antibiotics has led to a series of negative consequences, including the emergence of antibiotic-resistant bacteria and new pathogenic microorganisms, which urgently necessitate the development of new antibiotics to combat these threats (Kumar et al., 2023). Unfortunately, over the past 50 years, the discovery of new compounds has been infrequent (Wright, 2017). This may be attributed to the decreasing number of new strains available for study, as well as the obsolescence of current technologies and methods for natural product discovery, which require innovation (Hoffman, 2020).

Historically, Streptomyces has been the primary focus of antibiotic discovery research. However, recent studies have demonstrated that rare actinomycetes, such as *Nocardia* (Abdel-Razek et al., 2020) and *Amycolatopsis* (Adamek et al., 2018), represent a significant potential resource for secondary metabolites and have gradually emerged as key contributors to the discovery of new compounds. This shift has temporarily addressed the scarcity of strain resources in the search for novel compounds (Ezeobiora et al., 2022; Parra et al., 2023). Furthermore, research has revealed a substantial number of untapped BGCs within cultured actinomycetes. These abundant BGCs may serve as important resources for natural product discovery (Ding et al., 2019). However, experience and extensive research indicate that most of these BGCs remain silent, with only a limited number of compounds being identified from the relevant strains and BGCs. Consequently, traditional fermentation methods yield almost no corresponding secondary metabolites (Alam et al., 2023). To explore more natural products, various methods have been tried to activate silent gene clusters, including co-culture. For example, a series of phenazine-related compounds could be obtained by co-culturing *Micromonospora* sp. UR56 and *Actinokinespora* sp. EG49 (Hifnawy et al., 2020), two nonapeptide natural products were obtained by co-culture of *Amycolatopsis* sp. and *Tsukamurella pulmonis* (Pan et al., 2021). *Bacillus* sp. is also used to co-culture with other microorganisms and can stimulate another strain to produce secondary metabolites. Blennolide K can be obtained by co-culturing *Setophoma terrestris* with *B. amyloliquifaciens* (Arora et al., 2018); the co-culture of *Aspergillus versicolor* with *B. subtilis* produced four new compounds (Abdel-Wahab et al., 2019); two new compounds were produced in the co-culture of *Aspergillus ochraceuscolor* with *B. subtilis* (Frank et al., 2019); it is surprising that after co-culture of *Aspergillus sydowii* and *B. subtilis*, 25 new secondary metabolites were detected in the co-culture system (Sun et al., 2021). *Bacillus* species may be useful in co-culture, and co-culture may be a useful method to mine the related products of BGCs.

Determining strains that contain novel BGCs and have the potential to produce new compounds from a large number of actinomycetes has become an urgent task and a significant goal in the quest for discovering new natural products. In this study, we identified a novel BGC from *A. lurida* TRM64739 using the antiSMASH bacterial version tools (Kautsar et al., 2021; Navarro-Muñoz et al., 2020) and phylogenetic analysis. However, this BGC was silent under both experimental conditions and traditional fermentation methods in our initial experiments.

Subsequently, we co-cultured *A. lurida* TRM64739 with *Bacillus haynesii* in ISP3 liquid medium, successfully activating one of the iodinin-like BGCs, which led to the production of a series of phenazine compounds. Phenazine compounds are secondary metabolites with certain antibacterial activity produced by many microorganisms, especially bacteria. Phenazine compounds have been found antibacterial activity against *Staphylococcus aureus* (MRSA)TCH1516, and two-negative organisms: *Acinetobacter baumannii* 5075 and *Klebsiella pneumoniae* 1100 (Bauman et al., 2019), phenazine compounds also showed the cytotoxic activity (Viktorsson et al., 2021). This study marks the first successful application of BGCs analysis and co-culture methods to activate a silent BGC, achieving not only the targeted discovery of specific natural products but also providing a viable reference approach for the exploration of related natural products.

## 2 Materials and methods

### 2.1 Instrumentation

HPLC spectra were recorded on a Hitachi Primaide HPLC using an ODS column (YMC-Pack ODS-A, 10 × 250 mm, 5 μm, 3 mL/min) (YMC Co., Ltd.). LC-MS analysis was performed using an Acquity UPLC H-Class coupled to an SQ Detector 2 mass spectrometer, utilizing a BEH C18 column (1.7 μm, 2.1 × 50 mm, 1 mL/min) (Waters Corporation). NMR spectra were obtained using a Bruker AVANCE NEO 500 MHz spectrometer, an Agilent DD2-500 spectrometer, or a JEOL JNM-ECZ600R/S1 spectrometer, with tetramethylsilane (TMS) employed as an internal standard.

### 2.2 Culture conditions of the experiment strain

*A. lurida* TRM64739 and *B. haynesii* were established following their isolation from sediment samples collected from the Tarim River in China. The pathogenic bacteria utilized in the antagonistic tests were previously preserved within our research group. *A. lurida* TRM64739 was cultured and propagated on ISP4 solid medium at 30°C. Seed cultures were prepared in ISP4 liquid medium, while fermentation was conducted in oat liquid medium. In contrast, *B. haynesii* and the test pathogens were cultured in LB medium at 37°C. The composition of ISP4 medium (liquid/solid) includes: 10.0 g/L soluble starch, 1.0 g/L K_2_HPO_4_, 1.0 g/L MgSO_4_·7H_2_O, 2.0 g/L (NH_4_)_2_SO_4_, and 1.0 g/L NaCl, supplemented with 1.0 mL/L trace elements solution (composed of 0.1 g FeSO_4_·7H_2_O, 0.1 g MnCl_2_·4H_2_O, and 0.1 g ZnSO_4_·7H_2_O in 100.0 mL distilled water), adjusted to a pH of 7.5 ± 0.2. The composition of LB medium (liquid/solid) consists of 10 g/L tryptone, 5 g/L yeast extract, and 10 g/L NaCl, with a pH range of 7.0–7.4.

### 2.3 Analysis of BGCs in *Amycolatopsis lurida* TRM64739

The genome of the *A. lurida* TRM64739 was sequenced using Illumina HiSeq technology, and the complete genomic information was obtained following assembly. BGCs were identified using the online prediction analysis tool antiSMASH 7.0,^1^ which identifies BGCs using a signature profile Hidden Markov Model, Detection strictness: relaxed. The core and additional biosynthetic genes of the candidate BGCs were subjected to further analysis to select new BGCs and potential compounds. The iodinin-like BGC (Supplementary Table S1, Region 33.2) was compared with similar BGCs using phylogenetic methods.

### 2.4 Drug sensitive test

The drug sensitivity tests for *A. lurida* TRM64739 and *B. haynesii* were conducted using ISP4 and LB solid media, respectively. Rifamycin, chloramphenicol, kanamycin, and streptomycin were employed as antibiotics for the sensitivity assessments. The tests were performed utilizing the filter paper diffusion method. Antibiotics that inhibited the growth of *B. haynesii* but had no effect on *A. lurida* TRM64739 were subsequently selected as experimental antibiotics. The semi-inhibitory concentration of the antibiotic on *B. haynesii* was determined as the final co-culture concentration.

### 2.5 Activation of silent BGCs in *Amycolatopsis lurida* TRM64739

Seed liquid *A. lurida* TRM64739 was cultured on ISP4 solid medium at 30°C for 8 days to promote noticeable spore production and density. Subsequently, the bacterial cake (diameter = 0.5 mm) was inoculated into a triangular flask containing 150 mL of liquid ISP4 medium (in a 500 mL flask) and cultured at 30°C with shaking at 120 rpm for 5 days. Meanwhile, *B. haynesii* was cultured on LB solid medium at 37°C for 3 days, after which five bacterial cakes were inoculated into a triangular flask containing 150 mL of liquid LB medium (in a 500 mL flask) and cultured at 37°C with shaking at 120 rpm for 3 days.

Individual fermentation of *A. lurida* TRM64739 was conducted by inoculating a seed solution of TRM64739 into a triangular flask containing a liquid medium composed of oat, AM6, and millet (150 mL in a 500 mL flask) at an inoculation ratio of 1:15 (v/v). The culture was maintained at 30°C and agitated at 120 rpm for 10 days. Following fermentation, the broth was processed using macroporous resin (D101) and eluted with methanol, after which the secondary metabolites were analyzed by HPLC.

Preliminary test of co-culture: a total of 10 mL of TRM64739 seed solution and 2 mL of *B. haynesii* seed solution were inoculated into a triangular flask containing 150 mL of oat, AM6, and millet liquid medium (total volume of 500 mL). The culture was supplemented with 0.68 mg/L rifamycin and incubated at 30°C with shaking at 120 rpm for 10 days. Subsequently, the fermentation broth was processed using macroporous resin (D101), eluted with methanol, and the products were analyzed via HPLC. Large-scale co-culture: the TRM64739 and *B. haynesii* seed liquids were inoculated into a triangular flask (150 mL/500 mL) containing fermentation oat medium composed of 20 g/L oats, 1.0 g/L NaCl, 1.0 mL/L trace elements solution, and 0.68 mg/L rifamycin, adjusted to a pH of 7.5 ± 0.2. The volume ratios of TRM64739 seed liquid, *B. haynesii* seed liquid, and oat medium were maintained at 10:2:150, resulting in a total fermentation broth volume of 60 L.

### 2.6 Isolation and identification of the compounds from *Amycolatopsis lurida* TRM64739

The large-scale co-culture fermentation broth was subjected to macroporous resin (D101) and eluted with methanol, followed by drying under reduced pressure to obtain a crude extract. This extract was then separated using a silica gel column (100–200 mesh) and eluted with a dichloromethane/methanol system. The elution gradients employed were dichloromethane/methanol in the ratios of 1:0, 20:1, 10:1, 1:1, and 0:1, respectively. The fractions corresponding to the ratios of 1:0, 20:1, and 10:1 were further separated using gel columns (separated LH-20). The separation fractions were detected and combined under the guidance of HPLC, resulting in 10 fractions. Subsequently, five different compounds were purified by Prep-HPLC (preparative high performance liquid chromatography) from the Fr4, Fr7 and Fr8 (Supplementary Figure S1). The structures of each compound were identified using NMR and MS.

## 3 Results and discussion

### 3.1 Genome-mining of iodinin-like BGCs from *Amycolatopsis lurida* TRM64739

The antiSMASH analysis revealed that 43 BGCs were identified in the genome of *A. lurida* TRM64739, encompassing polyketide synthases (PKS), non-ribosomal peptide synthetases (NRPs), and PKS-like clusters, among others (Supplementary Table S1). Our findings indicate that strain TRM64739 harbors a substantial number of secondary metabolite gene clusters, which hold significant potential for the production of novel compounds. However, the newly predicted BGCs from the antiSMASH analysis include a considerable amount of unknown sequences (Navarro-Muñoz et al., 2020), and the delineation of these BGCs remains uncertain. Consequently, assessing the novelty of these BGCs and the structure of potential synthetic products is challenging.

We compared these BGCs with existing gene clusters in the MIBiG database and visualized the results using a phylogenetic tree. The core synthetic gene is critical to a BGC, and variations in this gene correspond to differences in the secondary metabolites produced.

TRM64739_33_Region2, as one of the BGCs, antiSMASH analyzed results showed that its most similar BGC is BGC0000935. Then, we retrieved nine other similar BGCs through the “Known Cluster Blast” function of the MIBiG database, according to the annotation of these BGCs in the MIBiG database, we downloaded and obtained the core genes of theses similar BGCs to TRM64739_3_Region2. The 11 core genes, including the core genes in TRM64739_33_ Region2, were used to construct the phylogenetic tree. The software was MEGA 7 and the method was neighbor-joining.

**Figure 1:**
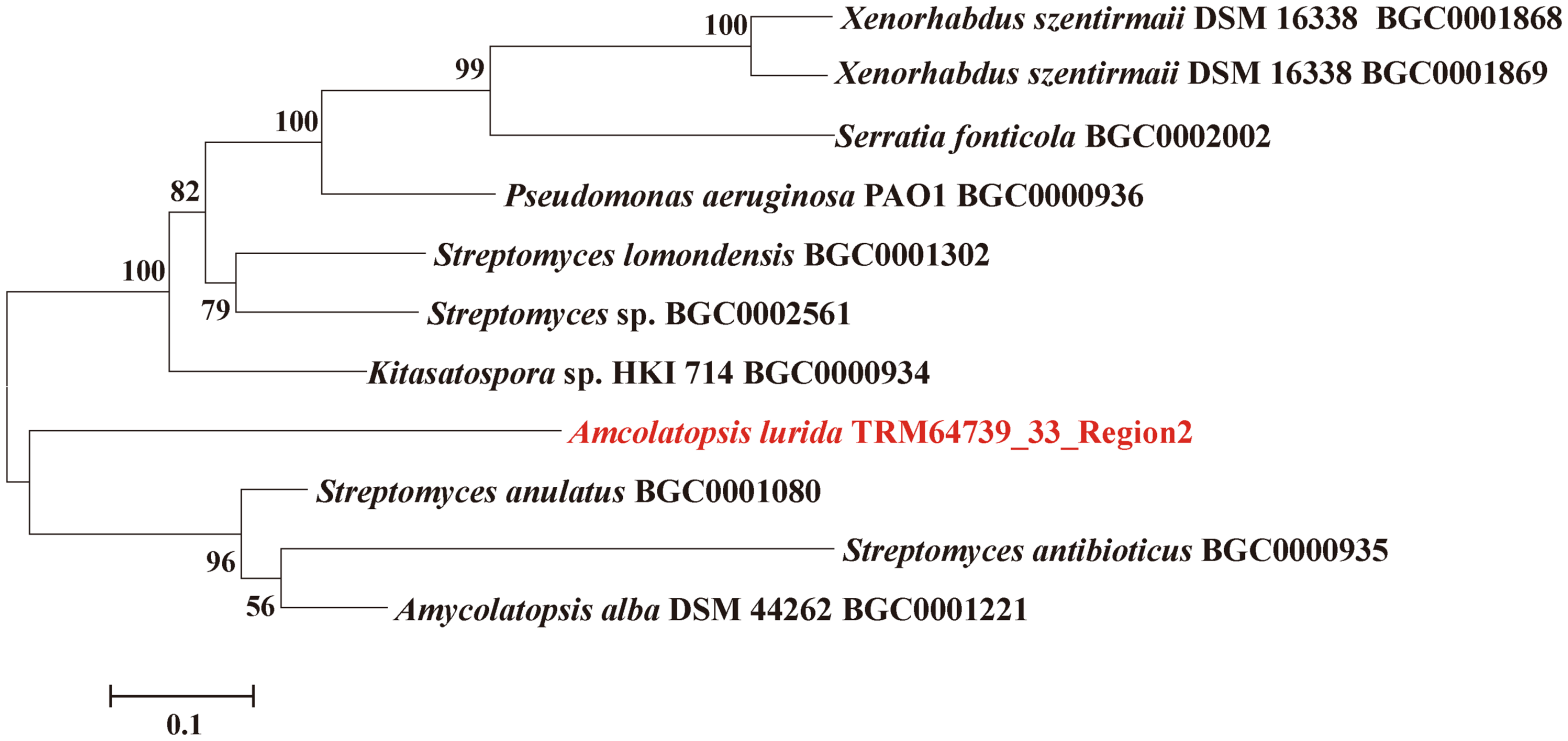
Neighbor-joining phylogenetic tree from the core genes of the TRM64739_3_Region2 and the similar BGCs constructed by MEGA 6. Numbers at nodes indicate levels bootstrap support (%) based on a neighbor-joining analysis of 1,000 resampled datasets; only values >50% are given.

The core synthetic gene of the iodinin-like BGC from the strain of TRM64739 was compared with 10 other BGCs using the phylogenetic tree. Notably, our interesting BGC formed an independent branch on the phylogenetic tree (TRM64739_33_Region2, Figure 1). Therefore, we speculate that the iodinin-like BGC in TRM64739 represents a novel BGC, and its corresponding secondary metabolites are likely to be new compounds that warrant further investigation.

### 3.2 Co-culture *Amycolatopsis lurida* TRM64739 with *Bacillus haynesii*

To obtain the products the novel biosynthetic gene clusters (BGC) in *A. lurida* TRM64739, pre-fermentation was conducted using oat, AM6, and millet liquid media. The secondary metabolites present in the fermentation broth were analyzed via high-performance liquid chromatography (HPLC). The results indicated that no significant secondary metabolites were detected in any of the three media. However, genomic analysis revealed that strain TRM64739 harbors a substantial number of secondary metabolite synthesis gene clusters. Consequently, we speculate that many of these gene clusters remain silent, suggesting that conventional fermentation methods are insufficient for the biosynthesis of these compounds.

**Figure 2:**
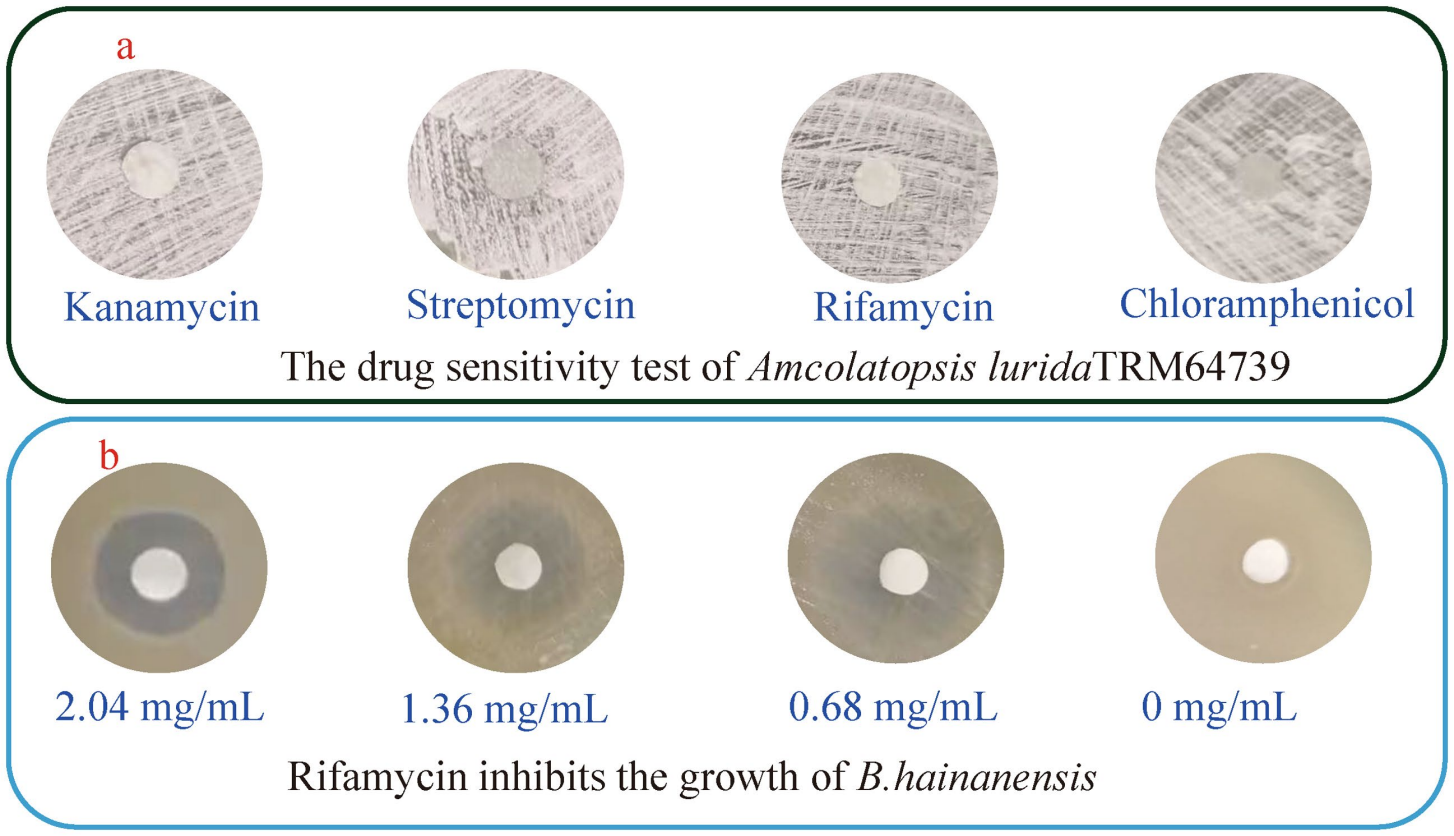
The drug sensitivity test of *A. lurida* TRM64739 and *B. haynesii*. **(a)** Sensitivity of *A. lurida* TRM64739 to streptomycin (2.21 mg/mL), rifamycin (2.04 mg/mL), kanamycin (1.98 mg/mL), and chloramphenicol (2.17 mg/mL). **(b)** Sensitivity of *B. haynesii* to rifamycin with the concentration as 20.4 mg/mL, 1.36 mg/mL, 0.68 mg/mL, and 0 mg/mL.

In order to activate the BGCs in the strain TRM64739 and obtain the desired response products, *A. lurida* TRM64739 was co-cultured with *B. haynesii*. However, *B. haynesii* exhibited a significantly faster growth rate than TRM64739 in oat, AM6, and millet media, resulting in a considerably higher final biomass. During the co-fermentation of *A. lurida* TRM64739 with *B. haynesii*, a “pollution” phenomenon was observed in the co-culture system. Consequently, controlling the growth rate and biomass of *B. haynesii* became crucial for the successful establishment of the co-culture system. It is well established that certain antibiotics can inhibit the growth of specific bacterial species while having no effect on others. An antibiotic will be identified that inhibits *B. haynesii* without affecting the growth of *A. lurida* TRM64739. Streptomycin (2.21 mg/mL), rifamycin (2.04 mg/mL), kanamycin (1.98 mg/mL), and chloramphenicol (2.17 mg/mL), were utilized in the drug sensitivity test for both *A. lurida* TRM64739 and *B. haynesii*, with the results displayed in Figure 2a. Rifamycin effectively inhibited the growth of *B. haynesii* while having no impact on the growth of *A. lurida* TRM64739. However, to facilitate co-culture, it is essential that the growth of *B. haynesii* is not entirely suppressed but rather maintained in a semi-inhibitory state. Therefore, we investigated the semi-inhibitory concentration of rifamycin for *B. haynesii*. As illustrated in Figure 2b, the results indicated that at a rifamycin concentration of 0.68 mg/mL, *A. lurida* TRM64739 and *B. haynesii* are most likely to achieve an optimal co-culture state. Under these conditions, the co-culture system should be successfully established.

**Figure 3:**
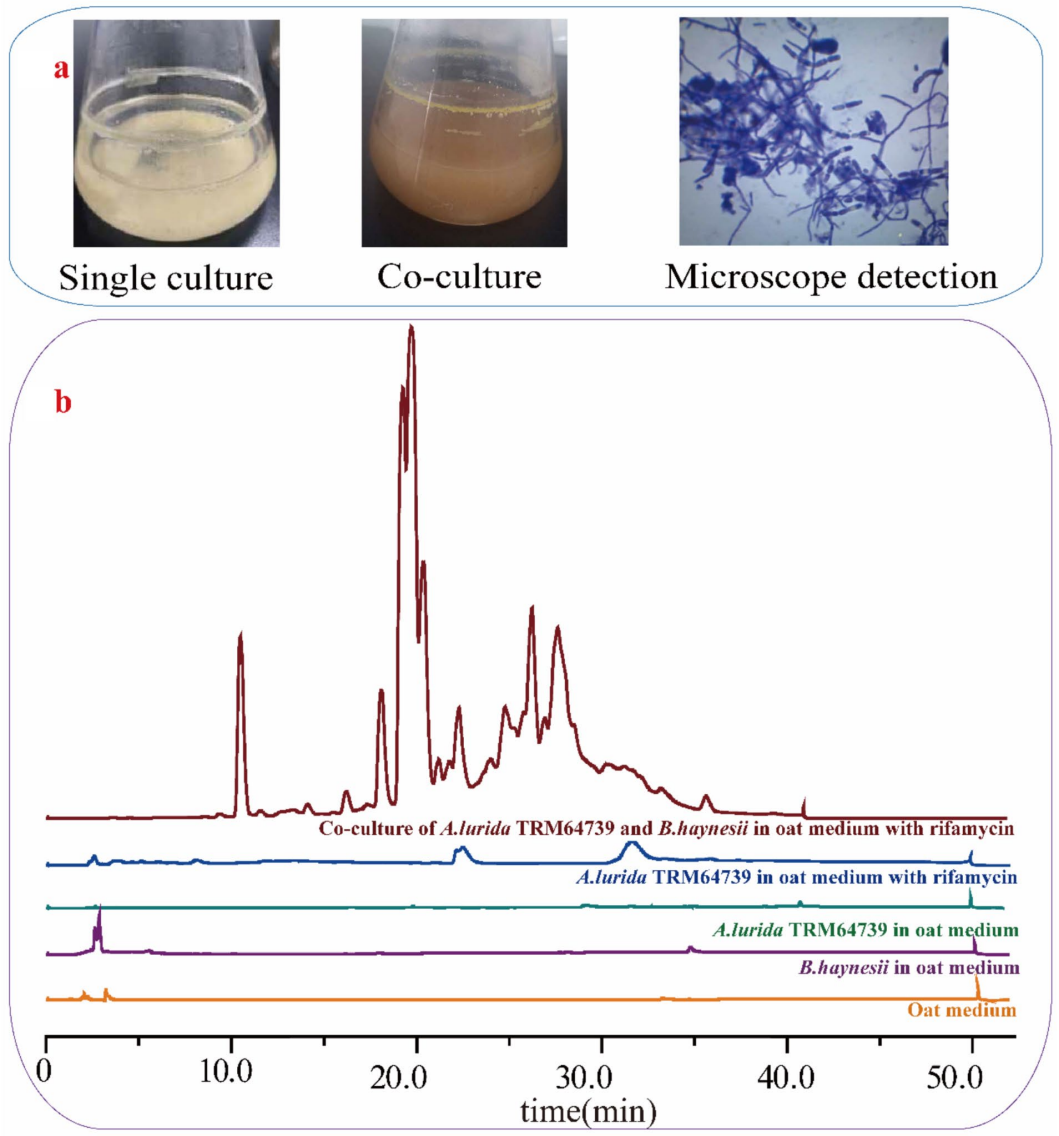
The co-culture of *A. lurida* TRM64739 with *B. haynesii*. **(a)** The appearance of *A. lurida* TRM64739 cultured alone and co-cultured with *B. haynesii* in oat medium. The growth state of *A. lurida* TRM64739 and *B. haynesii* in their co-culture system in oat medium (1,000-fold magnification). **(b)** HPLC detection results of secondary metabolites in five different fermentation broth from oat medium: oat medium (blank control), *B. haynesii* culured alone, *A. lurida* TRM64739 culured alone, *A. lurida* TRM64739 culured alone with rifamycin (0.68 mg/mL), *A. lurida* TRM64739 and *B. haynesii* co-culured with rifamycin (0.68 mg/mL).

*A. lurida* TRM64739 and *B. haynesii* were co-cultured in oat media, with the addition of 0.68 mg/mL rifamycin. The co-culture system is illustrated in Figure 3a. Following the addition of rifamycin, the growth of *B. haynesii* in the fermentation broth was effectively controlled. Under these conditions, *A. lurida* TRM64739 and *B. haynesii* established a favorable co-culture relationship. The fermentation broth from the co-culture was analyzed using HPLC to determine the production of secondary metabolites, with the results presented in Figure 3b.

In the co-culture conditions, *A. lurida* TRM64739 produced a greater quantity of secondary metabolites in oat fermentation media. This finding indicates that the silent BGCs in *A. lurida* TRM64739 were successfully activated due to the co-culture.

### 3.3 Separation and identification of products from *Amycolatopsis lurida* TRM64739

**Figure 4:**
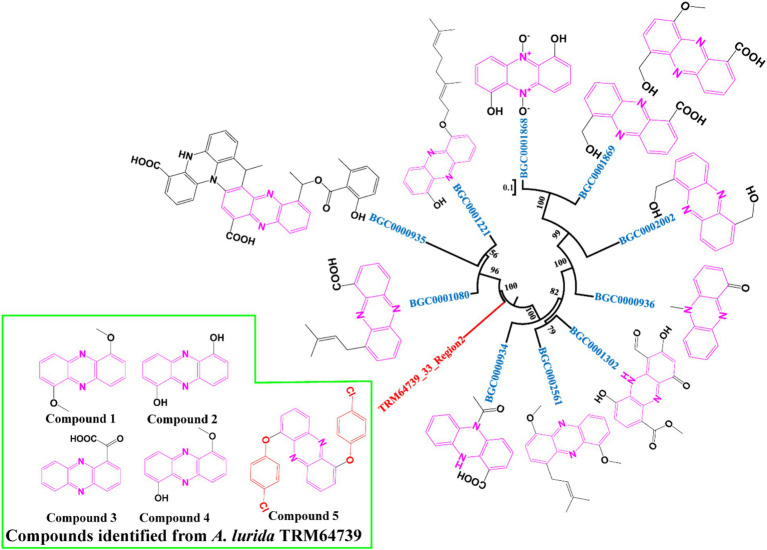
Neighbor-joining phylogenetic tree of BGCs synthesized phenazine compounds.

*A. lurida* TRM64739 was co-cultured with *B. haynesii* in an oat medium (70 L) supplemented with 0.68 mg/mL rifamycin. The fermentation broth was separated using macroporous resin (D101), silica gel column chromatography, and Prep-HPLC (Supplementary Figure S1, compounds 1–5). Ultimately, five phenazine compounds were identified (Figure 4). Compounds 1–4 are known, while compound 5 is a novel compound.

Compound 1 was a light-yellow solid, and the 1H NMR, 13C NMR, HSQC, and HMBC spectroscopic data (Supplementary Figures S3–S6 and Supplementary Table S2) were consistent with previous literature reports (Viktorsson et al., 2017). Therefore, compound 1 was identified as 1,6-dimethoxyphenazine (C_14_H_12_N_2_O_2_).

Compound 2 was a yellow solid, and the 1H NMR, 13C NMR, HSQC, and HMBC spectroscopic data (Supplementary Figures S8–S11 and Supplementary Table S3) were consistent with previous literature reports (Patel et al., 1984). Consequently, compound 2 was identified as 1,6-dihydroxyphenazine (C_12_H_8_N_2_O_2_).

Compound 3 was characterized as a yellow solid, and its 1H NMR, 13C NMR, HSQC, and HMBC spectroscopic data (Supplementary Figures S13–S16 and Supplementary Table S4) were consistent with previous literature reports (Jayatilake et al., 1996). Consequently, compound 3 was identified as phenazine-1-carboxylic acid (C_14_H_12_N_2_O).

Compound 4 was a light-yellow solid, and its 1H NMR, 13C NMR, HSQC, and HMBC spectroscopic data (Supplementary Figures S18–S21 and Supplementary Table S5) were consistent with previous literature reports (Lu et al., 2013). Consequently, compound 4 was identified as 6-hydroxy-1-methoxyphenazine (C_13_H_10_N_2_O_2_).

Compound 5 was characterized as a black-yellow solid, with its molecular formula deduced as C_24_H_14_N_2_Cl_2_O_2_ based on (+)-HR-ESI-MS analysis ([M + H] + calcd. 433.26137). In the 13C NMR spectrum (Supplementary Figure S25), six carbon signals were identified that correspond to the chemical shifts of 1,6-dihydroxyphenazine, along with an additional six carbon signals. Based on their chemical shifts, these latter signals were presumed to originate from aromatic rings, two of which (δC 124.8; δC 151.9) are not directly linked to hydrogen. It is hypothesized that the hydrogens at these positions may be replaced by chlorine and oxygen, respectively. Consequently, it is speculated that compound 5 results from the substitution of the hydroxyl hydrogen in iodinin (1,6-dihydroxyphenazine) by p-chlorobenzene (Figure 4, compound 5). The molecular weight (433.0) aligns with the results from (+)-HR-ESI-MS analysis (Supplementary Figure S23). Compound 5 was further characterized through detailed analyses of 1D and 2D NMR spectroscopy (Supplementary Figures S24–S27), as well as mass spectrometry data (Supplementary Figure S23), in conjunction with literature reviews, leading to the identification of compound 5 as a new structure, named it as 1,6-p-chlorophenylphenazine. Its 1H (500 MHz) and 13 C (125 MHz) NMR data was analyzed and organized in detail in Supplementary Table S6.

### 3.4 Inhibitory activity of compound-5 against six pathogens

**Figure 5:**
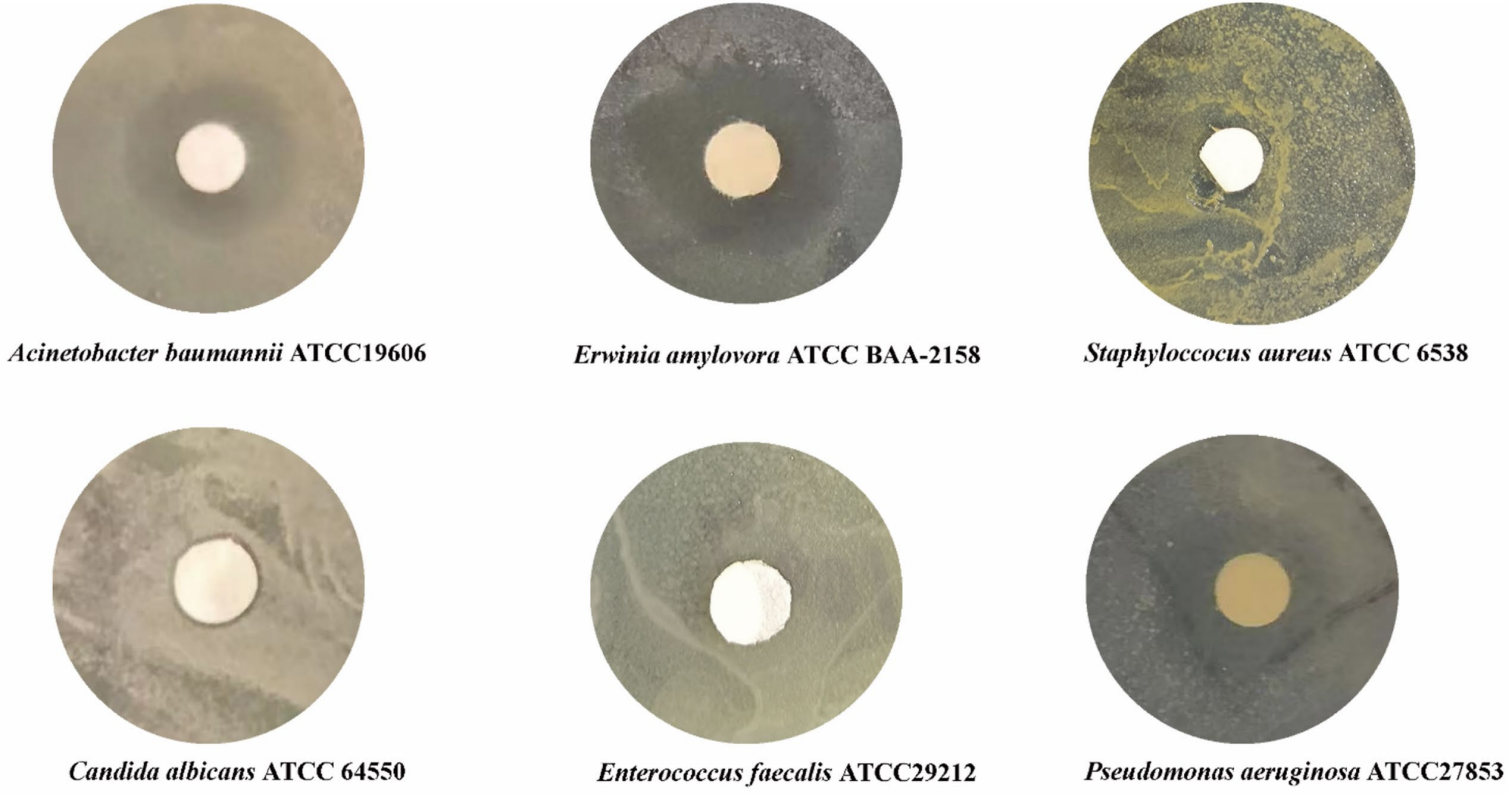
Inhibitory activity of compound 5 (1,6-p-chlorophenylphenazine) against six pathogens.

Given that compound 5 is a novel compound, it is essential to preliminarily investigate its inhibitory capacity. We selected six pathogens to evaluate its antibacterial activity. These pathogens include three Gram-negative bacteria (*Acinetobacter baumannii* ATCC19606, *Erwinia amylovora* ATCC BAA-2158, *Pseudomonas aeruginosa* ATCC27853), two Gram-positive bacteria (*Enterococcus faecalis* ATCC29212, *Staphylococcus aureus* ATCC 6538), and one fungus (*Candida albicans* ATCC 64550) (see Figure 5).

The filter paper (diameter: 0.3 mm) was soaked in a solution of compound 5 (0.36 mg/mL, solvent: DMSO), freeze-dried (−50°C, 9 pa), and sterilized by ultraviolet irradiation for antibacterial activity test. Six different pathogens were inoculated on the plate by coating inoculation method, and sterilized filter paper with compound 5 was placed on it. After 3 days of culture at 37°C, the bacteriostatic condition was observed.

Compound 5 exhibited no antibacterial activity against Gram-positive bacteria and fungi, but demonstrated significant antibacterial activity against Gram-negative bacteria (Figure 4), particularly against clinically drug-resistant strains (*A. baumannii* ATCC19606, *P. aeruginosa* ATCC27853) and plant pathogenic bacteria (*E. amylovora* ATCC BAA-2158). This suggests that the structure of compound 5 may provide a valuable reference for future drug development.

## 4 Conclusion

Numerous BGCs remain untapped in various actinomycetes, with many of them being silent (Kim et al., 2024). Co-culture has proven to be an effective and straightforward approach to activate these silent BGCs (Marmann et al., 2014; Selegato and Castro-Gamboa, 2023). In the co-culture system, one strain is often used to stimulate another strain containing a silent gene cluster to produce compounds. But what was the growth state and growth ratio of the two strains in the co-culture system, and whether it affected the activation of the silent gene cluster. Until 2017, a study of co-culture *Rhodococcus* sp. with *Mycobacterium* sp., an interesting experimental phenomenon was found, with the extension of culture time, the biomass of *Mycobacterium* sp. in the co-culture system is more and more, it may be that sufficient biomass provides a guarantee for *Mycobacterium* sp. synthesis products (Adnani et al., 2017). Mycolic acid had been considered to be the reason for stimulating the production of compounds by strains containing silent BGCs during co-culture. Onaka et al. (2011) found that it was the direct physical contact of the two bacteria rather than the mycolic acid that stimulated the strain to produce the compound in the co-culture of *S. lividans* and *T. pulmonis*. These results suggest that the production of mycolic acid would be not necessary for co-culture of bacteria. It also mean that there were more choices for bacteria for co-culture. Another noteworthy aspect is that it is necessary to ensure the growth of bacteria with the silent BGCs in the co-culture process.

However, in traditional co-culture experiments, the growth rate and biomass of the bacteria often exceed those of the actinomycetes. This issue can be effectively addressed by utilizing matched antibiotics at appropriate concentrations. Building on this premise, we employed bioinformatics analysis to identify novel BGCs in the strain TRM64739 and established a co-culture system involving *A. lurida* TRM64739 and *B. haynesii* by adding 0.68 mg/mL rifamycin. As a result, the silent BGCs were successfully activated, leading to the isolation and identification of five target compounds from the co-cultured fermentation broth. Notably, the new compound 5 isolated from *A. lurida* TRM64739 exhibited significant antibacterial activity against Gram-negative bacteria, highlighting its potential application value. This study presents a bioinformatics approach for mining novel BGCs from existing actinomycetes and demonstrates an effective method for activating silent BGCs to yield corresponding products via co-culture. The combined application of BGC analysis and co-culture strategies offers valuable insights and reference methodologies for future natural product discovery.

In previous research, the BGCs of *Amycolatopsis* species had been reported, it shows that *Amycolatopsis* species have strong potential for the synthesis of secondary metabolites. Including the strain of “*Amycolatopsis lurida* NRRL 2430” (Adamek et al., 2018), this strain has the same taxonomic status and Latin name as *A. lurida* TRM64739, but it has not been reported that *A. lurida* NRRL 2430 produces any secondary metabolites. Is this because of the lack of BGCs or the BGCs are silent in *A. lurida* NRRL 2430?

We sorted out the BGCs of *A. lurida* NRRL 2430 and found that there were only 26 BGCs (Supplementary Table S2), while *A. lurida* TRM64739 had 43 BGCs between *A. lurida* NRRL 2430 and *A. lurida* TRM64739 clusters, there were only nine analogous BGCs, indicating that although the two strains had the same taxonomic status, the BGCs and secondary metabolic potential were very different. This may be related to the habitat source of the strain, this result and phenomenon also suggests that the library of strains available for people to excavate natural products is more larger, and it also brings new hope for people to excavate more natural products.

Compound 5 (1,6-p-chlorophenylphenazine) exhibited antibacterial activity against drug-resistant strains (*A. baumannii* ATCC19606, *P. aeruginosa* ATCC27853) and *E. amylovora* ATCC BAA-2158, in the future, it may be used to control the diseases caused by these pathogens, or it may provide available lead compounds for the prevention and treatment of these pathogens. In addition, the antibacterial activity of compound 5 (1,6-p-chlorophenylphenazine) suggests that it may also play a role in the prevention and treatment of other related microbial diseases, which is worthy of follow-up research. Sousa et al. (2024) had made an in-depth analysis of the inhibitory activity mechanism of phenazine compounds. It is believed that phenazine compounds can inhibit the formation and maturation of biofilms by affecting the quorum sensing (QS) system and iron absorption mechanism of bacteria. Phenazine compounds can also be used as electron carriers to participate in the acquisition of iron, thereby affecting the formation of biofilms. Compound 5 (1,6-p-chlorophenylphenazine) is a phenazine compound, and its inhibitory activity may be also likely due to its action on biofilms.

## Data Availability

The original contributions presented in the study are publicly available. These data can be found in the NCBI under the following accession numbers: 16S rDNA sequence of Amycolatopsis lurida TRM64739 (PV539360), 16S rDNA sequence of Bacillus haynesii (PV539550), and genome sequence of Amycolatopsis lurida TRM64739 (JBNEBQ000000000).

## Footnotes

^1^ https://antismash.secondarymetabolites.org/#!/start
