# Supplementary material for: The taxonomy of Amycolatopsis lurida TRM64739 and Bacillus haynesii
Source: Front Microbiol. 2025 May 23;16:1571458. doi: 10.3389/fmicb.2025.1571458 (PMC12141291; doi:10.3389/fmicb.2025.1571458)
Supplement: Supplementary file 1 [file Data_Sheet_1.docx]

Supplementary Material

Discovery of Iodinin-like compounds by Co-Culture of *Amcolatopsis* *lurida* TRM64739 and *Bacillus haynesii*

Zhanwen liu, xinrong luo, zhanfeng xia, chuanxing wan, lili zhang

Table S1. The BGC annotation of *Amycolatopsis lurida*TRM64739

| Region | From | To | Known BGCs | Similarity |
| --- | --- | --- | --- | --- |
| [Region 1.2](#r1c2) | 632,193 | 654,763 | [Ery-9 / Ery-6 / Ery-8 / Ery-7 / Ery-5 / Ery-4 / Ery-3](https://mibig.secondarymetabolites.org/go/BGC0000513/1) | 100% |
| [Region 3.1](#r3c1) | 1 | 60,404 | [ansacarbamitocin A](https://mibig.secondarymetabolites.org/go/BGC0002011/1) | 6% |
| [Region 4.1](#r4c1) | 159,268 | 199,906 | [blasticidin S](https://mibig.secondarymetabolites.org/go/BGC0000874/1) | 7% |
| [Region 4.2](#r4c2) | 282,853 | 329,179 | [CC-1065](https://mibig.secondarymetabolites.org/go/BGC0001540/1) | 6% |
| [Region 5.1](#r5c1) | 14,592 | 272,165 | [nystatin-like Pseudonocardia polyene](https://mibig.secondarymetabolites.org/go/BGC0000116/1) | 56% |
| [Region 6.1](#r6c1) | 23,055 | 69,495 | [avilamycin A / avilamycin C](https://mibig.secondarymetabolites.org/go/BGC0000026/1) | 5% |
| [Region 7.1](#r7c1) | 130,067 | 265,207 | [ecumicin](https://mibig.secondarymetabolites.org/go/BGC0001582/1) | 10% |
| [Region 8.1](#r8c1) | 2 | 29,424 | [dechlorocuracomycin](https://mibig.secondarymetabolites.org/go/BGC0001569/1) | 8% |
| [Region 8.2](#r8c2) | 31,792 | 112,282 | [maytansine / ansamitocin P-3](https://mibig.secondarymetabolites.org/go/BGC0000020/1) | 12% |
| [Region 9.1](#r9c1) | 637 | 66,222 | [quartromicin A1](https://mibig.secondarymetabolites.org/go/BGC0000133/1) | 75% |
| [Region 9.2](#r9c2) | 121,957 | 239,280 | [amycolamycin A / amycolamycin B](https://mibig.secondarymetabolites.org/go/BGC0001503/1) | 38% |
| [Region 12.1](#r12c1) | 177,294 | 272,290 | [nocamycin](https://mibig.secondarymetabolites.org/go/BGC0001702/1) | 18% |
| [Region 13.1](#r13c1) | 87,848 | 180,629 | [sceliphrolactam](https://mibig.secondarymetabolites.org/go/BGC0001770/1) | 64% |
| [Region 14.1](#r14c1) | 62,745 | 84,400 | [brasilicardin A](https://mibig.secondarymetabolites.org/go/BGC0000632/1) | 46% |
| [Region 15.1](#r15c1) | 204,407 | 214,799 | [ectoine](https://mibig.secondarymetabolites.org/go/BGC0000853/1) | 100% |
| [Region 16.1](#r16c1) | 95,563 | 195,703 | [kendomycin](https://mibig.secondarymetabolites.org/go/BGC0001066/1) | 45% |
| [Region 17.1](#r17c1) | 15,019 | 56,776 | [ansacarbamitocin A](https://mibig.secondarymetabolites.org/go/BGC0002011/1) | 2% |
| [Region 19.1](#r19c1) | 1 | 14,996 | [fortimicin](https://mibig.secondarymetabolites.org/go/BGC0000695/1) | 6% |
| [Region 19.2](#r19c2) | 72,027 | 83,859 | [macrotetrolide](https://mibig.secondarymetabolites.org/go/BGC0000244/1) | 33% |
| [Region 19.4](#r19c4) | 172,059 | 205,997 | [auroramycin](https://mibig.secondarymetabolites.org/go/BGC0001522/1) | 4% |
| [Region 22.1](#r22c1) | 50,030 | 72,058 | [lankacidin C](https://mibig.secondarymetabolites.org/go/BGC0001100/1) | 26% |
| [**Region 24.1**](#r24c1) | **153,134** | **174,084** | [**isorenieratene**](https://mibig.secondarymetabolites.org/go/BGC0000664/1) | **42%** |
| [Region 26.1](#r26c1) | 8,572 | 29,660 | [daptomycin](https://mibig.secondarymetabolites.org/go/BGC0000336/1) | 7% |
| [Region 26.2](#r26c2) | 41,326 | 98,330 | [bleomycin](https://mibig.secondarymetabolites.org/go/BGC0000963/1) | 9% |
| [Region 32.1](#r32c1) | 90,384 | 133,842 | [albaflavenone](https://mibig.secondarymetabolites.org/go/BGC0000660/1) | 100% |
| [**Region 33.2**](#r33c2) | **109,767** | **135,349** | [**esmeraldin**](https://mibig.secondarymetabolites.org/go/BGC0000935/1) | **8%** |
| [Region 34.1](#r34c1) | 33,241 | 63,339 | [ansacarbamitocin A](https://mibig.secondarymetabolites.org/go/BGC0002011/1) | 4% |
| [Region 34.2](#r34c2) | 89,129 | 117,922 | [apoptolidin](https://mibig.secondarymetabolites.org/go/BGC0000021/1) | 38% |
| Region 35.1 | 31,372 | 113,101 | [amychelin](https://mibig.secondarymetabolites.org/go/BGC0000300/1) | 62% |
| [Region 38.1](#r38c1) | 6,678 | 88,414 | [RP-1776](https://mibig.secondarymetabolites.org/go/BGC0000429/1) | 8% |
| [Region 42.1](#r42c1) | 24,759 | 74,987 | [tiancimycin](https://mibig.secondarymetabolites.org/go/BGC0001378/1) | 5% |
| [Region 43.1](#r43c1) | 1 | 48,356 | [cytorhodin](https://mibig.secondarymetabolites.org/go/BGC0001568/1) | 3% |
| [Region 44.1](#r44c1) | 1,472 | 82,461 | [ulleungmycin](https://mibig.secondarymetabolites.org/go/BGC0001814/1) | 33% |
| [Region 50.1](#r50c1) | 30,503 | 55,809 | [amychelin](https://mibig.secondarymetabolites.org/go/BGC0000300/1) | 25% |
| Region | From | To | Known BGCs | Similarity |
| [Region 51.1](#r51c1) | 1 | 52,066 | [concanamycin A](https://mibig.secondarymetabolites.org/go/BGC0000040/1) | 28% |
| [Region 56.1](#r56c1) | 3,253 | 25,904 | [labyrinthopeptin A2 / labyrinthopeptin A1 / labyrinthopeptin A3](https://mibig.secondarymetabolites.org/go/BGC0000519/1) | 60% |
| [Region 59.1](#r59c1) | 1 | 29,467 | [incednine](https://mibig.secondarymetabolites.org/go/BGC0000078/1) | 9% |
| [Region 60.1](#r60c1) | 1 | 26,605 | [GE2270A](https://mibig.secondarymetabolites.org/go/BGC0000604/1) | 27% |
| [Region 62.1](#r62c1) | 1 | 21,755 | [trichamide](https://mibig.secondarymetabolites.org/go/BGC0000481/1) | 27% |
| [Region 64.1](#r64c1) | 1 | 17,132 | [halstoctacosanolide A](https://mibig.secondarymetabolites.org/go/BGC0000073/1) | 66% |
| [Region 76.1](#r76c1) | 1 | 7,983 | [chlorothricin / deschlorothricin](https://mibig.secondarymetabolites.org/go/BGC0000036/1) | 13% |
| [Region 84.1](#r84c1) | 1 | 4,706 | [niphimycins C-E](https://mibig.secondarymetabolites.org/go/BGC0001700/1) | 29% |

Table S2. The BGC annotation of *Amycolatopsis lurida NRRL 2430*

| Region | From | To | Known BGCs | Similarity |
| --- | --- | --- | --- | --- |
| Region 3.1 | 2 | 48,151 | mirubactin | 78% |
| Region 3.3 | 125,039 | 187,283 | albachelin | 100% |
| Region 4.2 | 152,479 | 172,731 | platencin | 12% |
| Region 4.3 | 203,255 | 245,885 | enduracidin | 8% |
| Region 4.4 | 257,542 | 278,504 | isorenieratene | 71% |
| Region 5.1 | 167,603 | 235,762 | dechlorocuracomycin | 12% |
| Region 5.2 | 262,020 | 300,435 | theonellamide | 26% |
| Region 6.1 | 32,721 | 43,110 | ectoine | 100% |
| Region 7.1 | 102,562 | 147,316 | arginomycin | 13% |
| Region 7.2 | 172,582 | 194,616 | lankacidin C | 20% |
| Region 9.2 | 147,284 | 203,827 | mayamycin | 72% |
| Region 10.1 | 1 | 13,891 | Ery-9 / Ery-6 / Ery-8 / Ery-7 / Ery-5 / Ery-4 / Ery-3 | 100% |
| Region 13.1 | 111,833 | 132,951 | vazabitide A | 4% |
| Region 14.1 | 43,245 | 64,174 | isorenieratene | 42% |
| Region 18.1 | 1 | 100,242 | ristomycin A | 100% |
| Region 19.1 | 21 | 49,646 | amycolamycin A / amycolamycin B | 31% |
| Region 22.1 | 1 | 22,394 | ansacarbamitocin A | 2% |
| Region 27.1 | 100,659 | 141,414 | kanamycin | 1% |
| Region 28.1 | 1 | 32,555 | lomofungin | 13% |
| Region 30.1 | 1 | 24,612 | yatakemycin | 6% |
| Region 30.2 | 89,388 | 132,150 | simocyclinone D8 | 8% |
| Region 31.1 | 2,965 | 44,116 | kinamycin | 5% |
| Region 33.1 | 5,927 | 27,057 | 2-methylisoborneol | 100% |
| Region 50.1 | 10,871 | 33,397 | citrulassin D | 100% |
| Region 55.1 | 9,187 | 50,311 | A54145 | 3% |
| Region 62.1 | 1 | 32,793 | coelibactin | 36% |

**
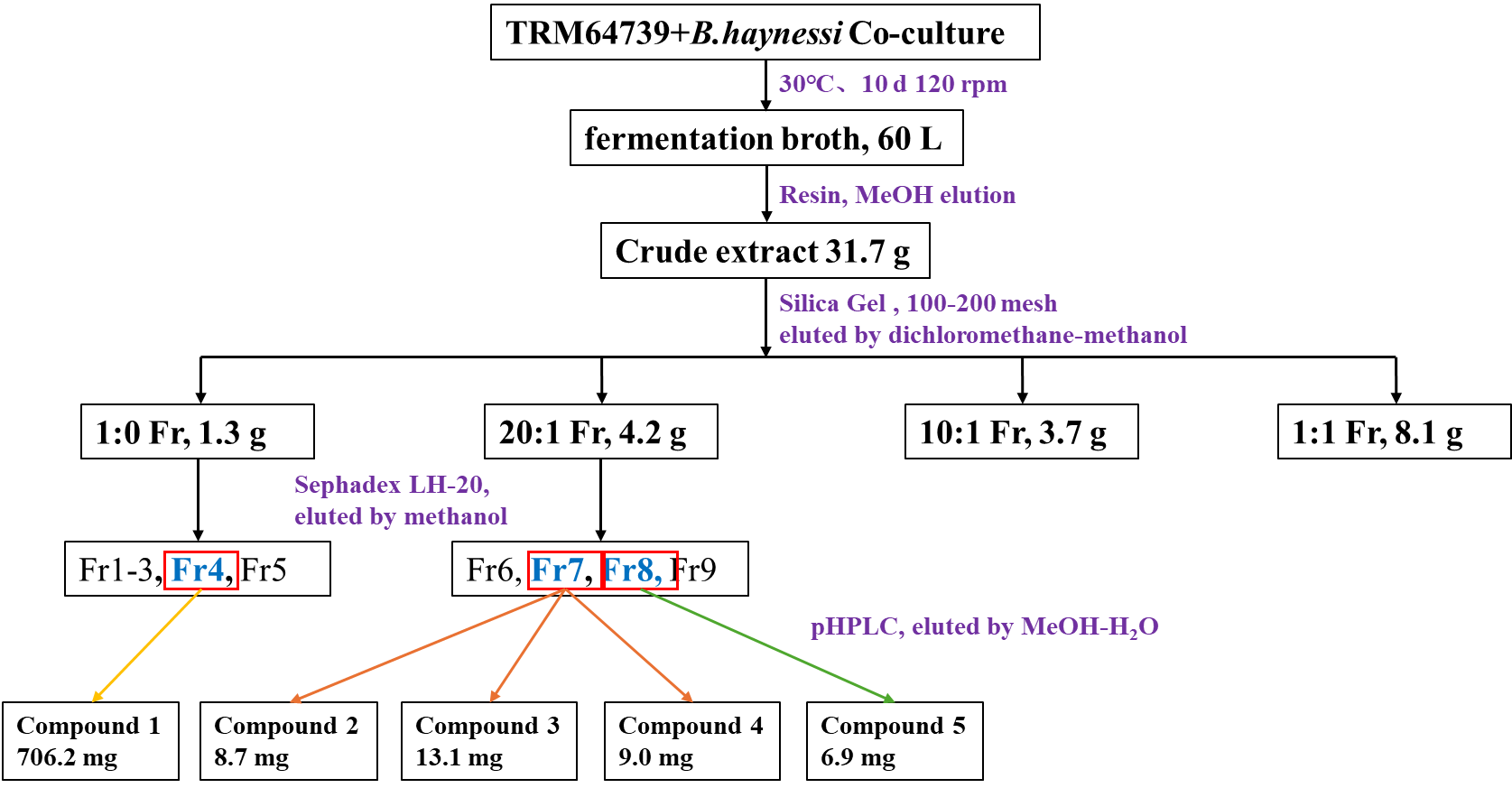
**

Figure S1. Compounds separated and identified from *Amcolatopsis* sp. TRM64739

Figure S2. Chemical structures of compound 1(1,6-Dimethoxyphenazine)，

“” HMBC relationship

Table S2. Summary of ^1^H ( 500 MHz) and ^13^ C ( 125 MHz) NMR data of compound 1

| Position | ^1^H NMR (δH, J in Hz) | ^13^C NMR (δC) |
| --- | --- | --- |
| 1 |  | 155.29 |
| 1` | 4.04（3H, s) | 56.49 |
| 2 | 7.27（1H, d,7.26) | 108.11 |
| 3 | 7.84（1H, dd,8.76,22.07) | 121.39 |
| 4 | 7.79（1H, d,7.26) | 131.28 |
| 4a | - | 142.80 |
| 5 | - | - |
| 5a | - | 136.20 |
| 6 |  | 155.29 |
| 6` | 4.04（3H, s) | 56.49 |
| 7 | 7.27（1H, dd,7.26) | 108.11 |
| 8 | 7.84（1H, dd,8.76,22.07) | 121.39 |
| 9 | 7.79（1H, d,7.26) | 131.28 |
| 9a | - | 142.80 |
| 10 | - | - |
| 10a | - | 136.20 |

**Figure S3**. ^1^H NMR (500 MHz, pyridine-*d*_5_) spectrum of compound **1**

**Figure S4**. ^13^C NMR (125 MHz, pyridine-*d*_5_) spectrum of compound **1**


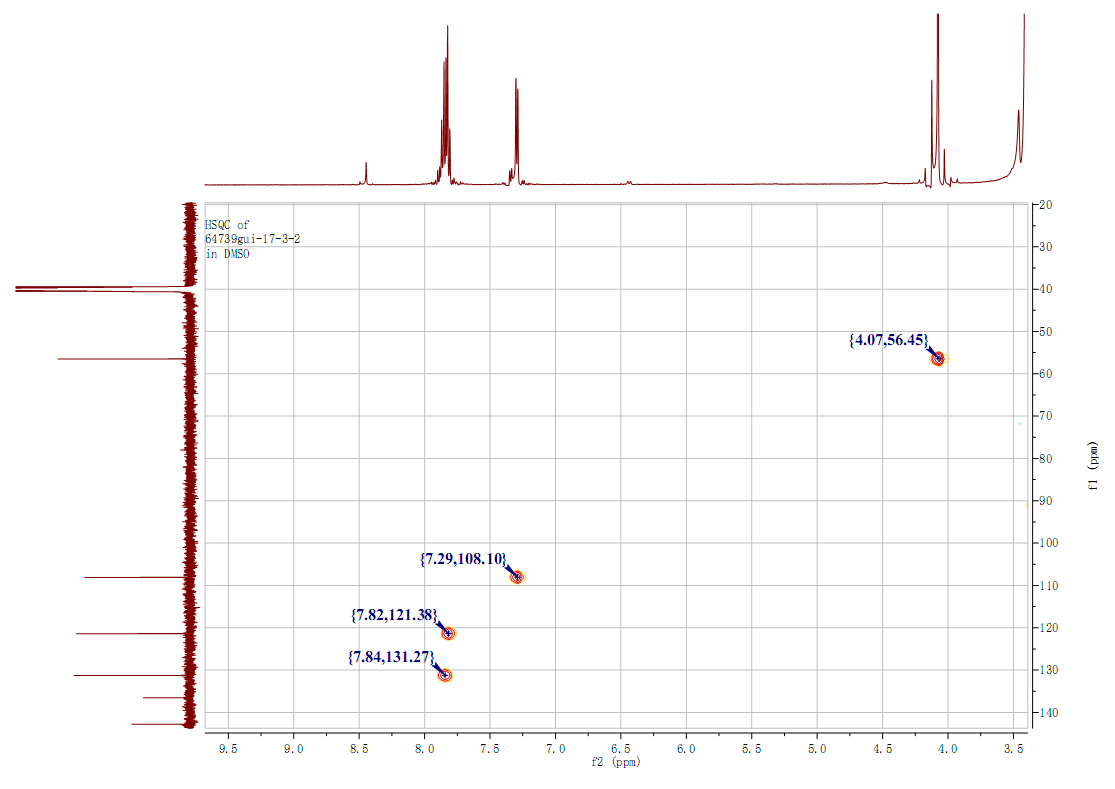


**Figure S5**. HSQC (pyridine-*d*_5_) spectrum of compound **1**


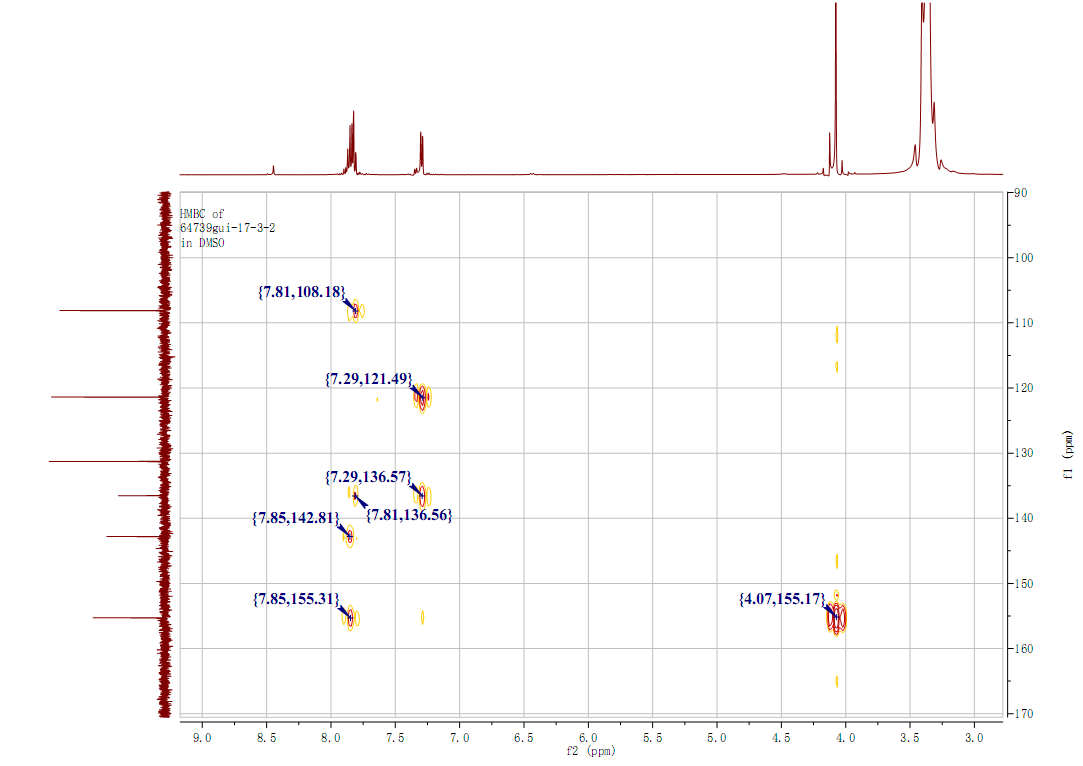


**Figure S6**. HMBC (pyridine-*d*_5_) spectrum of compound

**Figure S7**. Chemical structures of compound 2(1,6-dihydroxyphenazine)

Table S3. Summary of ^1^H ( 500 MHz) and ^13^ C ( 125 MHz) NMR data of compound 2

| Position | ^1^H NMR (δH, J in Hz) | ^13^C NMR (δC) |
| --- | --- | --- |
| 1 | - | 153.9 |
| 2 | 7.19（2 H,d 6.14） | 111.0 |
| 3 | 7.74（1 H, m, 7.72） | 131.8 |
| 4 | 7.74（1 H, m, 7.72） | 119.7 |
| 4a | - | 142.6 |
| 5 | - | -N |
| 5a | - | 136.2 |
| 6 | - | 153.9 |
| 7 | 7.19（2 H, d 6.14） | 111.0 |
| 8 | 7.74（1 H, m, 7.72） | 131.8 |
| 9 | 7.74（1 H, m, 7.72） | 119.7 |
| 9a | - | 142.6 |
| 10 | - | -N |
| 10a | - | 136.2 |
| -OH | 10.53（2H, brs, -OH） | - |

**Figure S8**. ^1^H NMR (500 MHz, pyridine-*d*_5_) spectrum of compound **2**

**Figure S9** ^13^C NMR (125 MHz, pyridine-*d*_5_) spectrum of compound **2**

**Figure S10** HSQC (pyridine-*d*_5_) spectrum of compound **2**

**Figure S11** HMBC (pyridine-*d*_5_) spectrum of compound **2**

Figure S12. Chemical structures of compound 3(phenazine-1-carboxylic acid)

Table S4. Summary of ^1^H ( 500 MHz) and ^13^ C ( 125 MHz) NMR data of compound 3

| Position | ^1^H NMR (δH，J in Hz) | ^13^C NMR (δC) |
| --- | --- | --- |
| 1 | - | 133.31 |
| 2 | 8.57（1H, d,6.50) | 134.59 |
| 3 | 8.54（1H, d,8.94) | 134.05 |
| 4 | 8.37（1H, dd,2.51,9.96) | 129.17 |
| 4a | - | 141.24 |
| 5 | - | - |
| 5a | - | 143.72 |
| 6 | 8.42（1H, dd,1.96,9.54) | 128.92 |
| 7 | 8.09（1H, m,5.29) | 132.551 |
| 8 | 8.11（1H, m,4.59) | 130.97 |
| 9 | 8.42（1H, dd,1.96,9.54) | 129.17 |
| 9a | - | 143.12 |
| 10 | - | - |
| 10a | - | 140.19 |
| 1` |  | 166.89 |

**Figure S13** ^1^H NMR (500 MHz, pyridine-*d*_5_) spectrum of compound **3**

**Figure S14** ^13^C NMR (125 MHz, pyridine-*d*_5_) spectrum of compound **3**

**Figure S15** HSQC (pyridine-*d*_5_) spectrum of compound **3**

**Figure S16** HMBC (pyridine-*d*_5_) spectrum of compound **3**

Figure S17. Chemical structures of compound 4(6-hydroxy-1-methoxyphenazine)

“” HMBC relationship

Table S5. Summary of ^1^H ( 500 MHz) and ^13^ C ( 125 MHz) NMR data of compound 4

| Position | ^1^H NMR (δH，J in Hz) | ^13^C NMR (δC) |
| --- | --- | --- |
| 1 | - | 155.4 |
| 2 | 7.22（1H, d,7.52) | 107.9 |
| 3 | 7.82（1H, d,9.0) | 131.8 |
| 4 | 7.82（1H, d,8.94) | 121.0 |
| 4a | - | 143.0 |
| 5 | - | - |
| 5a | - | 136.0 |
| 6 | - | 153.8 |
| 7 | 7.29（1H, d,5.70) | 111.3 |
| 8 | 7.77（1H, d,7.33) | 131.1 |
| 9 | 7.73（1H, d,8.16) | 121.4 |
| 9a | - | 142.2 |
| 10 | - | - |
| 10a | - | 136.8 |
| C,1-OCH_3_ | 4.07 (3H, s, 1-OCH3) | 56.5 |

**Figure S18** ^1^H NMR (500 MHz, pyridine-*d*_5_) spectrum of compound **4**

**Figure S19** ^13^C NMR (125 MHz, pyridine-*d*_5_) spectrum of compound **4**

**Figure S20** HSQC (pyridine-*d*_5_) spectrum of compound **4**

**Figure S21** HMBC (pyridine-*d*_5_) spectrum of compound **1**

Figure S22. Chemical structures of compound 5 (new compound)

Table S6. Summary of ^1^H ( 500 MHz) and ^13^ C ( 125 MHz) NMR data of compound 5

| Position | ^1^H NMR (δH，J in Hz) | ^13^C NMR (δC) |
| --- | --- | --- |
| 1 | - | 154.94 |
| 2 | 7.24(1 H, d 7.55) | 112.71 |
| 3 | 7.79(1 H, m, 7.75) | 132.58 |
| 4 | 7.91(1 H d, 9.31) | 107.57 |
| 4a | - | 144.64 |
| 5 | - | -N |
| 5a | - | 138.60 |
| 6 | - | 154.94 |
| 7 | 7.24(1 H, d 7.55) | 112.71 |
| 8 | 7.79(1 H, m, 7.75) | 132.58 |
| 9 | 7.91(1 H d, 9.31) | 107.57 |
| 9a | - | 144.64 |
| 10 | - | -N |
| 10a | - | 136.2 |
| 1` |  | 151.91 |
| 2` | 7.79(1 H, m 7.55) | 133.38 |
| 3` | 7.73(1 H, d 7.75) | 119.81 |
| 4` |  | 124.76(-Cl) |
| 5` | 7.09(1 H, d 7.10)112.59 | 112.59 |
| 6` | 7.79(1 H, m 7.75) | 133.36 |
| 1`` |  | 151.91 |
| 2`` | 7.79(1 H, m 7.55) | 133.38 |
| 3`` | 7.73(1 H, d 7.75) | 119.81 |
| 4`` |  | 124.76(-Cl) |
| 5`` | 7.09(1 H, d 7.10)112.59 | 112.59 |
| 6`` | 7.79(1 H, m 7.75) | 133.36 |


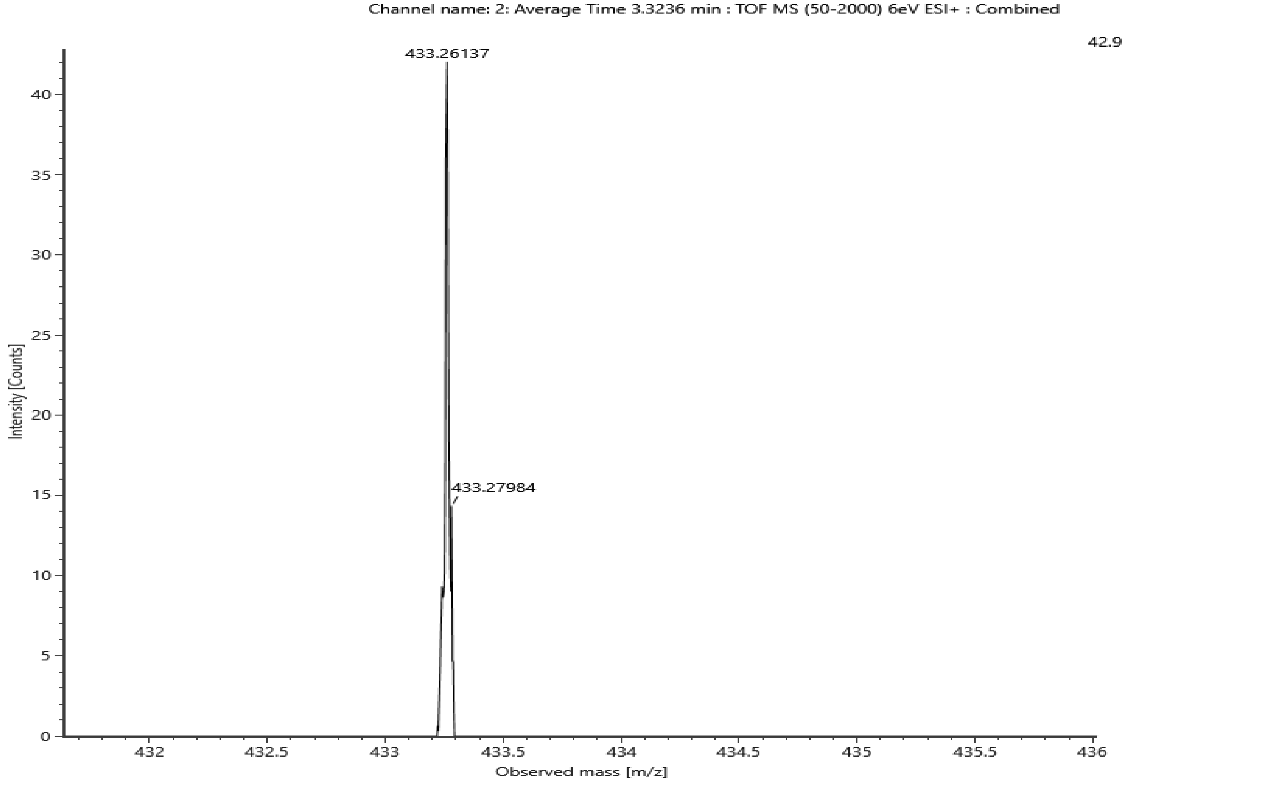


**Figure S23. HR-ESI-MS spectrum of compound 5**

**Figure S24** ^1^H NMR (500 MHz, pyridine-*d*_5_) spectrum of compound **5**

**Figure S25** ^13^C NMR (125 MHz, pyridine-*d*_5_) spectrum of compound **5**

**Figure S26** HSQC (pyridine-*d*_5_) spectrum of compound **5**

**Figure S27** HMBC (pyridine-*d*_5_) spectrum of compound **1**
